# Impact of Market Access Delays on Time to Patient Access: Multi-Country Comparative Analysis Assessing the First Commercial Launch Indications for Five Oncology Medicines Across Europe and Canada

**DOI:** 10.3390/jmahp14020025

**Published:** 2026-04-28

**Authors:** Barry Crean, David Parry, Alison Horsfield, James Ryan, Nektarios Oraiopoulos

**Affiliations:** 1 Pharmaceutical Sciences, R&D, AstraZeneca, Cambridge CB2 0AA, UK; 2 Oncology Market Access and Pricing, Oncology Business Unit, AstraZeneca, Cambridge CB2 1RY, UK; 3 Cambridge Judge Business School, University of Cambridge, Cambridge CB2 1AG, UK

**Keywords:** health technology assessment, market access process, life-years lost, time to patient access, oncology medicines, regulatory approval, reimbursement, survival benefit

## Abstract

**Background:** The benefit of pharmaceutical innovation manifests when patients access treatment. Following regulatory approval in Europe and Canada, reimbursement decisions depend on health technology assessments (HTAs), which can be prolonged. To quantify the impact of delays on patients, we evaluated market access timelines for olaparib, osimertinib, durvalumab, acalabrutinib, and trastuzumab deruxtecan across six high-income countries with established HTA systems (Canada, England, France, Germany, Italy, Spain). **Methods:** Time to access was from regulatory approval to reimbursement. Survival benefit was median overall survival (OS) and progression-free survival (PFS) assessed versus the comparator at approval and the latest data cut-off. The number of eligible patients per year multiplied by the years to patient access and survival benefit reflects the lost survival benefit. **Results:** Efficacy benefits observed at approval continued to the latest data cut-offs. The mean time to patient access was 18 months. Although this varied by country and treatment, with England and Germany typically being the fastest and France and Spain the slowest, timelines often exceeded the 180-day EU target despite identical evidence used in HTA submissions. This resulted in an estimated mean of 2836 patients being unable to access treatment and 3391 OS-derived and 2739 PFS-derived life-years lost. **Conclusions:** Access processes must evolve to ensure the timely realization of new medicines’ benefits.

## 1. Introduction

The value of pharmaceutical innovation is only realized when patients access treatment. The process for a medicine to reach patients involves multiple coordinated steps. Following regulatory agency approval (Health Canada in Canada, European Medicines Agency [EMA] in Europe, Medicines and Healthcare products Regulatory Agency [MHRA] in the UK), health technology assessment (HTA) bodies then assess a range of evidence, including the clinical evidence, comparative effectiveness, and economic value of the medicine and issue a recommendation on therapeutic benefit and cost-effectiveness (HTA benefit assessment outcome) [1]. Following successful negotiations with payers, the medicine achieves reimbursement listing and is funded for prescribing. Patients can only routinely access the medicine once this process is complete.

Market access processes differ across countries. Indeed, although EMA approval is centralized, market access remains the responsibility of individual countries, meaning that new medicines do not become accessible to patients across Europe at the same time.

For instance, HTA agencies in France and Germany [2,3] review the clinical data using comparative clinical effectiveness assessments, which include the efficacy, patient outcomes, and safety of the assessed medicine compared with the current standard of care. In Germany, new oncology medicines are reimbursed and become available for patient access immediately after EMA approval, in parallel with the full HTA process and price negotiations. HTA agencies in Canada [4] and England [5] formally assess the economic evidence in addition to the clinical evidence by considering the expected additional clinical benefit relative to the additional cost of introducing a new medicine compared with the current standard of care using cost-effectiveness assessments. HTA agencies may also assess the budget impact of new medicines, evaluating how their costs affect the overall healthcare or pharmaceutical budget beyond what is already allocated.

In oncology, intensive research has led to the development of many innovative treatments, such as the cancer medicines olaparib [6,7,8,9,10], osimertinib [11,12,13,14,15,16], durvalumab [17,18,19], acalabrutinib [20,21,22], and trastuzumab deruxtecan [23,24,25,26], which are the topic of this manuscript. These medicines all demonstrated efficacy during their clinical development and received regulatory approval. In this study, we refer to the point at which regulatory approval and national reimbursement have both been achieved as “launch”. Patients often face prolonged delays before the market access process concludes [27]. One reason for this is that data on long-term survival, a key focus for payers, are often unavailable and/or immature at the time of launch [28,29]. This requirement for long-term survival data affects evidence availability for early-stage disease, chronic cancers, and treatments with curative intent. For instance, neither median overall survival (mOS) nor median progression-free survival (mPFS) was reached for acalabrutinib monotherapy in first-line treatment of chronic lymphocytic leukemia at the time of regulatory approval and the HTA submission because this disease progresses slowly [20]. This means that the long-term benefit of treatment is uncertain. Although this is not an issue for regulators who accept progression-free survival (PFS) as meaningful, payers are more risk-averse because of the potential lost opportunity cost. Immature overall survival (OS) data can therefore slow the HTA process and hinder payer decision-making [28,29,30]. In addition, there are process inefficiencies caused by the overlap between regulatory evaluations and HTA agencies; for example, both authorities review the clinical data for a medicine, usually sequentially, which may contribute to delays in patient access [31,32].

This study aimed to assess market access in Canada, England, France, Germany, Italy, and Spain for the first launches of five AstraZeneca cancer medicines (olaparib, osimertinib, durvalumab, acalabrutinib, and trastuzumab deruxtecan). Countries with established HTA systems were selected from the World Bank high-income category to ensure comparable economic development [33,34]. Although market access processes differ across countries, as explained above, they are sufficiently similar to allow for meaningful comparison [35]. Canada was selected for two reasons. First, similar to many European countries, it has a well-established HTA system [34]. Second, although Canada has a national HTA, decision-making occurs at the sub-regional (provincial) level, which aligns with the vision of the Joint Clinical Assessment (JCA) in the EU, a recent collaborative assessment conducted by multiple EU member states to evaluate the clinical evidence of new medicines and reduce duplication [36,37]. This approach enabled us to assess the impact of HTA-related delays on patient access and outcomes across Europe and Canada, while also identifying whether particular market access processes were associated with longer delays.

The five oncology medicines were chosen for assessment because they are often a healthcare priority for countries, given the associated high unmet need and disease severity. Moreover, all five medicines were made by a single manufacturer (AstraZeneca) and underwent their first launch indications in Europe and Canada during the study time frame. Finally, these five medicines represent different types of development scenarios and clinical evidence, such as Phase III trials, single-arm trials and trials with immature data.

The overall aim of this study was to quantify the time from regulatory approval to patient access across economically developed countries with different market access processes and how these timings may have impacted patient outcomes.

## 2. Methods

### 2.1. Data Sources

This study assessed the market access of five AstraZeneca cancer medicines in six countries for their first indications from December 2014 to August 2024. The study cut-off date was 31 August 2024, defined prior to the preparation of this manuscript. At this time, HTAs for trastuzumab deruxtecan in this clinical indication had not been submitted in Canada or Italy and had received a negative recommendation in Spain; this is noted in the analyses.

Olaparib, osimertinib, durvalumab, acalabrutinib, and trastuzumab deruxtecan were evaluated in their first launched indications in Europe and Canada. These indications and the mOS and mPFS values used in the HTA submissions are described in Table 1 (see also Table S1 for a more comprehensive overview, which includes trial numbers, completion date, and links to the webpages of the trials).

Table 2 summarizes the data sources used to inform this analysis. Information was obtained from several sources. These included peer-reviewed publications, regulatory assessment reports and proprietary databases. The data gathered covered clinical efficacy, key clinical trials and data cut-offs used for regulatory and HTA evaluations, epidemiological estimates and access milestones (such as regulatory approval and reimbursement listings).

Table 3 summarizes the HTA agencies and early access programs to provide context for differences in patient access timing across countries. Early access programs enable patients with high unmet needs to receive new medicines prior to completion of the full HTA and reimbursement process. Germany is the only country with a formal process that funds treatment immediately from EMA approval.

Reimbursement listing dates were taken from the NAVLIN database (https://data.navlin.com/alspc/#!/, accessed on 13 October 2025) using the data sources listed in Table S3. There were no conflicting milestones identified across data sources; therefore, heterogeneity of data sources and hierarchy of data did not affect the analyses.

### 2.2. Outcomes Evaluated for First Launch

All data sources used to calculate outcomes are summarized in Table 2. All calculations for each medicine in each country were performed using Microsoft Excel, and the mean of these results was subsequently derived and presented.

This analysis is descriptive and is intended to illustrate differences in market access timelines across countries rather than establish causal relationships. All data are presented in aggregate rather than at the patient level, because the analysis focuses on the overall societal impact rather than individual-level outcomes.

#### 2.2.1. Time to Patient Access

Time to patient access was calculated as the time interval between regulatory approval and reimbursement listing for the first launched indication for each medicine, expressed in months. The numbers used to obtain the results are reported in Table S4. For any treatments that were not submitted for reimbursement, a data cut-off date of 31 August 2024 was used. In addition, the following were also evaluated: time from regulatory approval to HTA benefit assessment outcome and time from HTA submission to HTA benefit assessment outcome.

#### 2.2.2. Survival Benefit

Two survival benefit outcomes were calculated for each medicine in the first launched indication: the difference in mPFS and mOS between the new medicine versus the control arm (OS- and PFS-derived survival benefit, respectively). Indirect comparison methods were used to quantify the survival benefit for the single-arm trials (SATs) DESTINY-Breast01 [25], AURA2 [12], and AURAex [16]. For DESTINY-Breast01, a matching-adjusted indirect comparison (MAIC) [50] was used to compare trastuzumab deruxtecan with data from external comparator studies [25]. Individual patient data from DESTINY-Breast01 were weighted to align baseline characteristics with those of patients in comparator trials. This enabled indirect comparisons of treatment effects to be made, based on the assumption that all relevant prognostic factors and effect modifiers were adequately adjusted for in the model [25,51]. For the AURA2 and AURAex trials [12], a synthetic control arm from the IMPRESS [48] study was used to evaluate osimertinib. This synthetic arm combined external trial data to provide a comparator arm, allowing indirect estimation of treatment benefit. Clinical survival benefit data for each medicine at the time of launch and at the most recent follow-up were compared to assess if the benefit observed at the time of the regulatory submission was maintained over time.

#### 2.2.3. Lost Clinical Survival Benefit

The number of eligible patients who could not access treatment because of reimbursement process timelines was calculated as follows: number of patients affected = incidence in country × time interval from marketing application approval to product listing. Additional information on epidemiology data is available in Table S5.

The lost clinical survival benefit was calculated as follows: number of OS- or PFS-derived years lost = incidence in country × OS or PFS benefit × time interval from marketing application approval to product listing. This approach is in line with previously published methods [52,53].

As a scenario (sensitivity) analysis, outcomes were re-estimated assuming reimbursement listing within 6 months of regulatory approval, which aligns with the 180-day EU target for timely patient access [54]. Where subtracting 6 months would yield an implausible interval, a minimum lag of 1 month was applied for calculation purposes.

## 3. Results

### 3.1. Time to Patient Access

Table 4 presents the comparative therapeutic benefit assigned to the first indications of the included medicines in Germany and France, in which HTA frameworks formally evaluate and publish these assessments. It shows that the extent of added benefit varied across medicines and was, in some cases, revised upon resubmission when mature OS data became available.

All treatments were reimbursed in all markets, except for trastuzumab deruxtecan, which was not submitted for HTA in Canada or Italy and received a negative HTA recommendation from the HTA body in Spain. Olaparib initially received a negative recommendation in Canada based on Study 19 data but was subsequently accepted based on the primary results from SOLO-2 [9,10]. Similarly, the osimertinib HTA initially received a negative recommendation in Germany based on the two SATs AURAex [11,16] and AURA2 [12], but it was subsequently accepted based on the health-related quality of life (HRQoL) and safety results of the AURA3 RCT [13,15]. The HTA recommendations from the first submission in all countries assessed are shown in Table 5. These were categorized as positive, negative, or positive with restrictions, where restrictions indicate approval limited to specific conditions such as a narrower patient population or defined clinical criteria.

**Figure 1:**
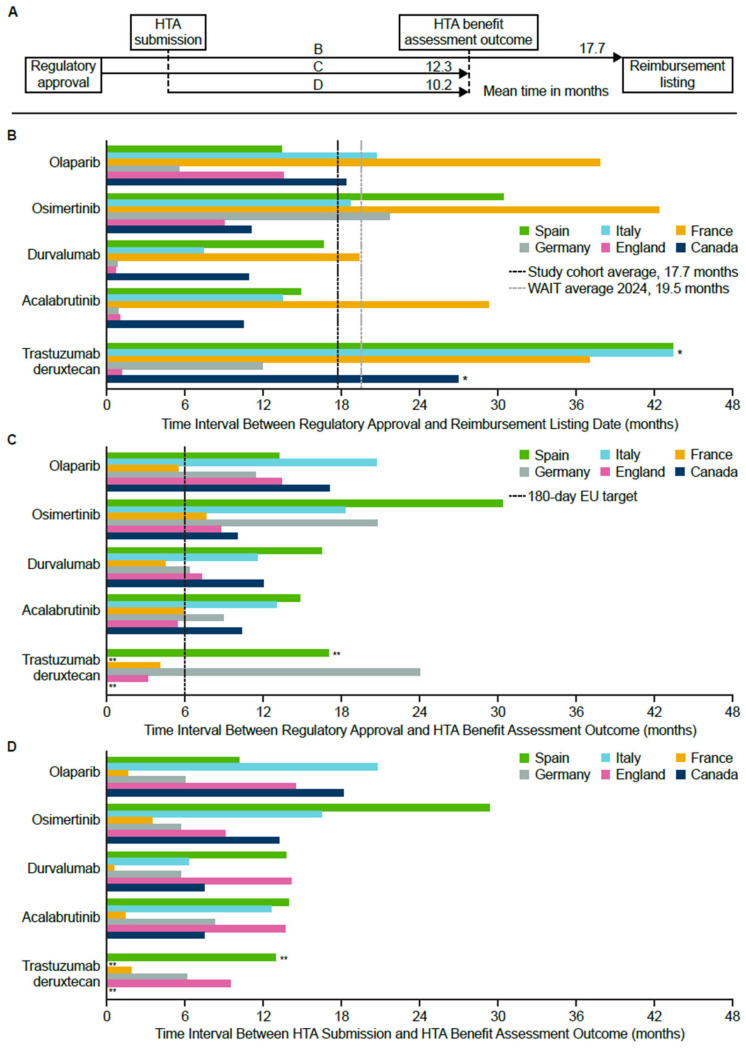
(**A**) Schematic of the market access process and estimated mean timings of the study cohorts, where B represents the mean time between regulatory approval and reimbursement listing, C the mean time between regulatory approval and HTA benefit assessment outcome, and D the mean time between HTA submission and HTA benefit assessment outcome. Note: the schematic uses a different scale (e.g., 1 cm = 1 month) compared with the timelines shown in Figure 1B–D. (**B**) Time to patient access defined as the interval from regulatory approval to reimbursement listing. (**C**) The interval from regulatory approval to HTA benefit assessment outcome. (**D**) The interval from HTA submission to HTA benefit assessment outcome. * HTA was not submitted for trastuzumab deruxtecan in Canada and Italy; ** The trastuzumab deruxtecan indication was not launched in Canada, Italy, and Spain. EU = European Union; HTA = health technology assessment; WAIT = Waiting to Access Innovative Therapies [55].

Across medicines and countries, the mean time to patient access, defined as the time from regulatory approval to product listing, was 17.7 months (Figure 1B and Table S4). England and Germany typically had the shortest intervals, while France and Spain had the longest. Of note, most cases exceeded the 180-day EU target [54]. Trastuzumab deruxtecan was assessed using a pivotal SAT (DESTINY-Breast01). SATs are commonly used in populations with high unmet need and limited treatment options, but because they lack a randomized comparator, they introduce greater uncertainty than an RCT. In this case, submissions based primarily on the SAT DESTINY-Breast01 with comparative effects estimated via a series of MAICs contributed to delays in reimbursement decisions in several countries, illustrating how evidence type can directly influence the speed of patient access to innovative cancer therapies. Figure 1A shows a schematic of the mean timelines of the study cohorts described in the bar graphs. Analyzing the components of time to access, the mean time from regulatory approval to HTA benefit assessment outcome was 12.3 months (excluding trastuzumab deruxtecan in Canada and Italy; Figure 1C and Table S4), which was considerably greater than the 180-day EU target. In addition, the mean time from HTA submission to HTA benefit assessment outcome was 10.2 months (excluding trastuzumab deruxtecan in Canada and Italy; Figure 1D and Table S4).

### 3.2. Survival Benefit

**Figure 2:**
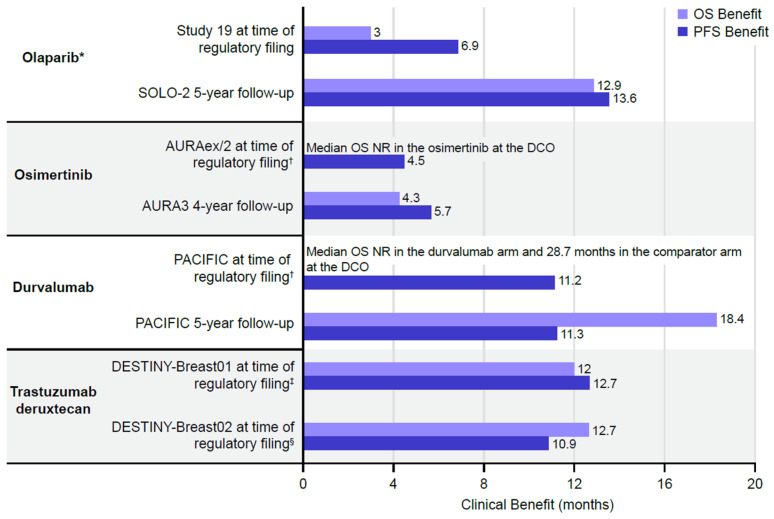
OS and PFS benefit. * Olaparib survival benefit data shown for *BRCA*-mutated patient population. ^†^ AURAex/2 pooled analysis of single-arm trials. IMPRESS synthetic control arm used to calculate PFS benefit. ^‡^ MAICs were used to calculate survival benefit. ^§^ Long-term follow-up data unavailable at time of publication. DCO = data cut-off; MAIC = matching-adjusted indirect comparison; mOS = median overall survival; mPFS = median progression-free survival; OS = overall survival. mOS and mPFS were not reached at the time of regulatory filing or latest DCO for acalabrutinib; therefore, acalabrutinib was excluded from the analysis.

The mOS and mPFS benefits (i.e., the differences between treatment and control arms) observed for the first launched indications continued to the most recent data cut-off for all medicines except acalabrutinib, for which mOS and mPFS were not reached at either timepoint (Figure 2 and Table 6). Durvalumab did not reach the mOS at the primary data cut-off, and acalabrutinib did not reach the mOS or mPFS at either the primary data cut-off or the long-term follow-up. Figure 2 is provided for illustrative purposes only because absolute changes in survival benefit between first and confirmatory studies for a given medicine have not been quantified owing to baseline differences between studies, such as patient populations, lines of prior treatment, and extent of crossover (e.g., patients in the control arm switching to the experimental treatment after disease progression, which was allowed in the clinical trials assessing olaparib, osimertinib, and acalabrutinib). In the case of durvalumab, the same PACIFIC Phase III trial was used at regulatory submission (primary analysis) and long-term follow-up; here, there was no meaningful change in mPFS benefits from the regulatory approval (11.2 months) to long-term 5-year follow-up (11.3 months) [17,18,19]. OS was longer than PFS, which likely reflects the durable immune-mediated effect of durvalumab, with some patients deriving continued benefit beyond disease progression, as observed in the PACIFIC trial and other studies [17,19,56].

### 3.3. Lost Clinical Survival Benefit

**Figure 3:**
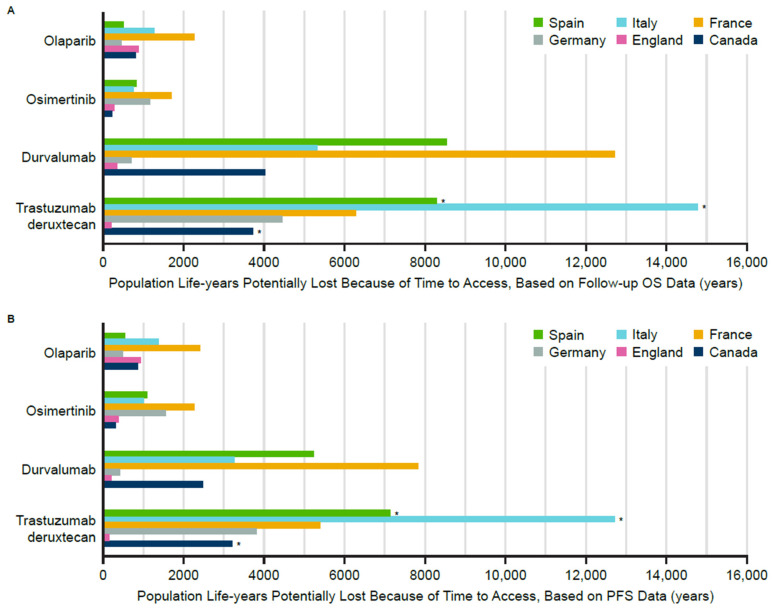
Population life-years lost from delayed access, (**A**) OS-derived life-years lost and (**B**) PFS-derived life-years lost. * The trastuzumab deruxtecan indication was not launched in Canada, Italy and Spain. Median OS and median PFS were not reached at the time of regulatory filing or latest DCO for acalabrutinib, and therefore acalabrutinib was excluded from the analysis. DCO = data cut-off; OS = overall survival; PFS = progression-free survival.

An estimated average of 2836 patients could not access treatment because of delays during the time from regulatory approval to reimbursement (Figure 3). The lowest number of patients who could not access treatment was observed for acalabrutinib (127 patients in England), and the highest for trastuzumab deruxtecan (13,986 patients in Italy). Moreover, 3391 OS-derived life-years and 2739 PFS-derived life-years (Figure 3 and Table 6) were potentially lost on average per medicine per country during the time from approval to reimbursement. Among the five countries, France and Italy had the highest number of life-years lost on average (France: 5774 OS-derived and 4494 PFS-derived life-years; Italy: 5569 OS-derived and 4610 PFS-derived life-years), while England had the lowest number lost on average (468 OS-derived and 461 PFS-derived life-years). Among all medicines, the greatest mean number of life-years was lost for trastuzumab deruxtecan (6320 OS-derived life-years), while the lowest average number of life-years was lost for osimertinib (857 OS-derived life-years).

It is noteworthy that while the ELEVATE-TN trial met its endpoints for PFS (PFS was the primary endpoint for acalabrutinib in combination with obinutuzumab and the secondary endpoint for acalabrutinib monotherapy), mOS and mPFS were not reached at either the interim analysis used in the regulatory submission or the final trial analysis after 6 years of follow-up [57]. Consequently, lost clinical survival benefit could not be derived for acalabrutinib from ELEVATE-TN in this analysis.

### 3.4. Sensitivity Analysis

As a scenario analysis, we recalculated outcomes assuming access occurred within 6 months of regulatory approval (Table 7). This shortened the mean approval-to-access interval by 12.9 months and reduced the estimated number of patients unable to access treatment and the corresponding OS- and PFS-derived life-years lost (by 2071, 2457, and 2017, respectively). These findings support the internal validity of the approach, showing that earlier access reduced delays, life-years lost, and the number of patients without access by more than 70%.

## 4. Discussion

This study demonstrates the impact on patients and families of delayed access to oncology medicines while awaiting long-term survival data. The mean time to patient access, from regulatory approval to reimbursement listing, was 18 months. Although access times varied by country and treatment, with England and Germany typically taking the shortest time and France and Spain the longest, they often exceeded the 180-day EU target, despite identical evidence being used in HTA submissions. These delays translate directly into lost life-years, as reflected in our calculations of overall survival- and progression-free survival-derived life-years lost. By focusing on measurable patient outcomes, this study underscores how delays in HTA and reimbursement compromise timely access to potentially life-saving treatments. These delays may also contribute to wider societal costs, including avoidable healthcare utilization (e.g., hospitalization) and productivity losses due to early loss of employment.

The time from regulatory approval to HTA recommendation exceeded the 180-day EU target [54] in all five countries for at least one treatment, with three EU countries (France, Italy and Spain) and Canada exceeding this target for all five treatments. While Canada is not bound by EU regulations, the 180-day EU target is widely recognized as an international benchmark for timely patient access. Given the structural similarities between Canada’s HTA system and those in Europe, this standard provides a meaningful point of comparison for evaluating HTA timelines across all included countries [34,37].

The observed average time from regulatory approval to patient access of 17.7 months highlights the impact on patients and families, with wider societal implications, consistent with Waiting to Access Innovative Therapies indicator survey data reporting an average of 19.5 months for oncology medicines in 27 EU countries in 2024 [55]. Overall, although there was variability in time from regulatory approval to HTA recommendation across countries, differences in market access processes did not consistently explain why some countries experienced longer or shorter delays than others.

England and Germany had some of the fastest times from regulatory approval to reimbursement listing; however, in Germany, medicines submitted based on SATs (e.g., osimertinib) had considerably longer times, illustrating how different countries’ approaches to evidence type can influence access timelines.

France had some of the longest times due to extended pricing and reimbursement negotiations, with only a short interval from HTA submission to assessment outcome (average 2 months) but an average of 27 months from assessment outcome to reimbursement listing.

Following regulatory approval, delays in HTA initiation and lengthy assessment processes often reflect differing evidence requirements across HTA bodies and regulators, particularly regarding long-term outcomes (Table 8). HTA agencies may prioritize certainty and magnitude of clinical benefit, favoring mature or statistically robust data, which can delay recommendations. Delays in oncology medicine market access directly affect patient outcomes, with patients in some countries waiting over a year for access, potentially reducing survival and HRQoL benefits. Importantly, some patients may never receive the medicine in time to experience these benefits. Conditional reimbursement can mitigate this when benefit is uncertain, such as with SATs, by allowing access while collecting additional evidence.

Trastuzumab deruxtecan illustrates this issue. Countries accepting SATs with indirect comparisons, such as England, granted quick access. Countries not accepting such comparisons, like Canada and Germany, effectively delayed submission until comparative trial data were available. This led to approximately a one-year delay in patient access in Germany (February 2022 vs. March 2021) and continued unavailability in Canada, limiting access to patients unless they pay privately.

Variations in times to patient access are driven by more than differences in HTA evidence requirements. Table 8 summarizes biases from the perspective of various stakeholders that may influence decision-making and affect time to patient access. These biases (e.g., information bias driven by the requirement for certainty before decision-making) provide further context for differences in time to patient access observed across countries and highlight how factors can shape decision-making at both the individual and organizational levels. Prolonged regulatory and market access processes may place particular pressure on smaller biotech companies, which play a major role in driving innovation. When these developers cannot achieve timely, successful launches, the resulting risk of market failure can ultimately deny patients access to innovative medicines. These factors also reflect differences in the timing and availability of information across stakeholders, which can contribute to inefficient decision-making and delayed patient access. Overall, this affects how quickly patients gain access to treatment, helping explain why countries using the same evidence may still move at different speeds and how this contributes to the wider societal impact of delayed access. Together, these factors highlight the importance of approaches that can better manage clinical uncertainty and support earlier patient access, including early access programs and stronger partnering across stakeholders.

Early access programs exist to reduce wait times, although Germany is the only country with a formal process that systematically funds treatment immediately from EMA approval. France has a publicly funded early access program [58]; England uses the Cancer Drug Fund [59]. For the first launch indications of the medicines evaluated (not including subsequent indications), over 12,000 patients in England and France benefited from these programs. Other countries use temporary classification or immediate launch funding that allow limited or interim access to patients, which is different from Germany, where medicines can be reimbursed immediately at launch while assessment and pricing negotiations occur.

A limitation of this study is that the OS- and PFS-derived life-years lost may be under- or overestimated, for example, owing to treatment crossover during clinical trials, which can dilute observed overall survival benefits. In addition, the date of reimbursement listing may be less meaningful from a patient perspective, as patients may seek access as soon as positive data become publicly available (e.g., following presentations at major oncology congresses). Access delays after reimbursement (ranging from 2 months in England to 1 year in Italy or Spain) [55], including diffusion of medicines into routine clinical practice, may not be fully captured, which may underestimate delayed access. Conversely, excluding early access programs, compassionate use, private funding, and clinical trial availability may overestimate lost life-years. Patient factors such as performance status, personal choice, attrition, and access to oncologists may also influence how many patients ultimately receive the medicine, meaning that the number of patients treated in practice may be lower than the number considered eligible. On balance, these analyses provide an illustrative estimate of the patient impact of delays, with mature, controlled OS data likely to represent the most robust indicator.

In our scenario analysis, we recalculated outcomes assuming access occurred within 6 months of regulatory approval, which aligns with the 180-day EU target for timely patient access [54]. The results showed that meeting this target would have reduced access delays, the number of patients without access, and OS- and PFS-derived life-years lost by more than 70%, highlighting the potential benefits for patients if the 180-day target is achieved.

In addition, this study focuses on survival outcomes (and losses) using OS/PFS estimates; however, it is important to acknowledge that delays in access and the associated lack of PFS benefit can contribute to early clinical deterioration, with increased care needs, which can impact patients, families, carers, and the wider healthcare system. This is reflected in intermediate endpoints such as progression-free survival, which capture worsening disease and increasing care needs well before mortality. However, exploring this factor in more detail was outside the scope of this study.

Finally, although the included countries have comparable levels of economic development, differences in their market access processes, criteria, and timelines limit the generalizability of the findings across countries.

Despite these limitations, the analysis provides a clear, quantitative assessment of the consequences of delayed access to innovative oncology treatments on patient outcomes in Europe and Canada. These analyses are intended as illustrative, population-level indicators of potential impact, rather than precise patient-level causal effects, because median OS and PFS values from clinical trials represent summary measures rather than individual survival predictions and do not reflect the variability in eligibility, uptake, and access patterns seen in clinical practice.

## 5. Conclusions

The average time from regulatory approval to reimbursement was 17.7 months for five cancer medicines across six countries, with the time from EMA approval to HTA recommendation substantially exceeding the 180-day EU target across all countries for most treatments.

The duration from HTA benefit assessment outcome to reimbursement listing was substantial and varied.

The market access process led to approximately 3000 life-years potentially lost for patients who could not access these medicines. As drug development increasingly targets earlier and curative-intent settings, long-term outcome uncertainty will likely exacerbate these access challenges. Early disease detection and treatment benefits will only be realized if patients can access therapies. Despite centralized EMA approval, national market access processes mean patients across Europe do not receive new medicines at the same time. Multiple indications for any given medicine under evaluation further complicate HTAs because they require consideration of differential value. The EU JCA [36] is a collaborative assessment conducted by multiple EU member states to evaluate the clinical evidence of new medicines and reduce duplication. EU JCA may help to streamline market access processes and improve adherence to the 180-day EU target, provided that countries use the EU JCA submission and report, reduce national process timelines when the clinical assessment has already been undertaken, or start their market access process earlier. All stakeholders should urgently consider partnering to improve patient access, particularly for promising new medicines with uncertain long-term benefits.

## Figures and Tables

**Table 1:** Overview of the medicines evaluated in the study: indications at first major launch and supporting trials.

Medicine	Indication	Study Name and Design	N	Control Arm	OS/PFS (Months)
**Olaparib** **PARP inhibitor**	Platinum-sensitive relapsed ovarian, fallopian tube, or primary peritoneal cancer [12,38]	Study 19 [6,7,8,39] Phase II randomized	265	Placebo	ITT population: OS 29.8 vs. 27.8; PFS 8.4 vs. 4.8 BRCA mutation subgroup: mOS 34.9 vs. 31.9; mPFS 11.2 vs. 4.3
		SOLO-2 [9,10] Phase III randomized	295	Placebo	OS 51.7 vs. 38.8; PFS 19.1 vs. 5.5
**Osimertinib** **EGFR tyrosine kinase** **inhibitor**	EGFR T790M+ NSCLC [40,41]	AURAex [11,16] Phase I/II single-arm	201	None	OS NR; PFS 11
		AURA2 [12] † Phase II single-arm	210	None	OS NR; PFS 9.9
		AURA3 [13,15] † Phase III randomized	419	SoC	OS 26.8 vs. 22.5; PFS 10.1 vs. 4.4
**Durvalumab** **Anti–PD-L1 monoclonal antibody (immune checkpoint inhibitor)**	Stage III NSCLC (post-CRT) [42,43]	PACIFIC [17,18,19] Phase III randomized	713	Placebo	OS 47.5 vs. 29.1; PFS 16.9 vs. 5.6
**Acalabrutinib** **Bruton’s tyrosine** **kinase** **inhibitor**	Treatment-naïve and relapsed/refractory CLL [44,45]	ELEVATE-TN [20,21,22] Phase III randomized	535	SoC	OS NE; PFS NE
**Trastuzumab** **deruxtecan** **HER2-directed antibody–drug conjugate**	HER2+ metastatic breast cancer [46,47]	DESTINY-Breast01 [25] Phase II single-arm	184	None	OS 24.6; PFS 16.4
		DESTINY-Breast02 [23] Phase III randomized	599	SoC	OS 39.2 vs. 26.5; PFS 17.8 vs. 6.9

*BRCA* = breast cancer gene; CLL = chronic lymphocytic leukemia; CRT = chemoradiotherapy; EGFR = epidermal growth factor receptor; HER2 = human epidermal growth factor receptor 2; ITT = intention-to-treat; mOS = median overall survival; mPFS = median progression-free survival; NE = not evaluable; NR = not reached; NSCLC = non-small cell lung cancer; OS = overall survival; PARP = poly(ADP-ribose) polymerase; PD-L1 = Programmed Death-Ligand 1; PFS = progression-free survival; SoC = standard of care. Primary endpoints were met in all trials. More details on how all efficacy endpoints were assessed in each trial can be found in the source studies referenced in this table and on the https://www.clinicaltrials.gov/ webpages of each trial. † The data used in this study to calculate OS and PFS benefit are from a pooled analysis of AURA2 and AURAex (mOS = NR and mPFS = 4.5).

**Table 2:** Summary of data sources used to calculate time to patient access, survival benefit, and lost clinical survival benefit.

Parameter	Source
Clinical efficacy data (OS and PFS)	Published articles [6,8,9,10,11,12,13,14,15,16,17,19,20,22,23,24,25,26,39,48,49]
Regulatory assessment of clinical benefit–risk balance	Published European Public Assessment Reports (EMA) and Summary Basis of Decision (Health Canada)
Epidemiology	© 2025 DR/Decision Resources, LLC *
Payer assessment for clinical benefit–risk balance	IQVIA HTA Accelerator disease database ^†^
Regulatory approval dates	
Reimbursement listing dates	NAVLIN database ^‡^

EMA = European Medicines Agency; HTA = health technology assessment. All data were extracted from the databases reported in this table and compiled in Microsoft Excel for analysis. * All rights reserved. Reproduction, distribution, transmission, or publication is prohibited. Reprinted with permission. The database is available via paid access from https://clarivate.com/life-sciences-healthcare/portfolio-strategy/market-assessment/epidemiology-intelligence/ (accessed on 5 January 2023). ^†^ The database contains published data provided by each country’s health authorities and is available online via paid access from www.iqvia.com/landing/hta-accelerator (accessed on 18 February 2023). ^‡^ The database contains published data provided by each country’s health authorities and is available online via paid access from https://data.navlin.com (accessed on 28 February 2024). Additional information on data sources is available in Table S2.

**Table 3:** Summary of HTA agencies and early patient access programs in the different countries.

Country	Accountable HTA Agency	Early Patient Access Overview
Canada	CDA-AMC (https://www.cda-amc.ca/) (accessed on 13 October 2025)	No publicly funded early access program
England	NICE (https://www.nice.org.uk/) (accessed on 13 October 2025)	Publicly funded early access program for unserved patient population or high unmet need within approved label
France	HAS (https://www.has-sante.fr/) (accessed on 13 October 2025) Five levels of clinical benefit compared with alternative comparative therapy are assigned, which influence pricing; levels I–IV indicate increasing degrees of added clinical benefit, while level V indicates no added benefit	
Germany	G-BA (https://www.g-ba.de) (accessed on 13 October 2025) Six levels of clinical benefit compared with alternative comparative therapy are assigned, which influence pricing; levels 1–4 indicate increasing degrees of added clinical benefit of decreasing magnitude, level 5 indicates no added benefit, and level 6 indicates less benefit than the comparator	Access granted from EMA approval
Italy	AIFA (https://www.aifa.gov.it/) (accessed on 13 October 2025)	No publicly funded early access program
Spain	AEMPS (https://www.aemps.gob.es/) (accessed on 13 October 2025)	

AEMPS = Spanish Agency of Medicines and Medical Products; AIFA = Italian Medicines Agency; CDA-AMC = Canada’s Drug Agency–Agence des Médicaments du Canada; EMA = European Medicines Agency; G-BA = German Federal Joint Committee; HAS = French National Authority for Health; HTA = health technology assessment; NICE = National Institute for Health and Care Excellence.

**Table 4:** Overview of benefit assessments taken from the HTA submission for the first indication of the presented medicines.

Medicine	Germany	France
Olaparib	IV: non-quantifiable added benefit III: minor added benefit on resubmission with RCT data	IV: minor therapeutic improvement
Osimertinib	V: no added benefit II: considerable added benefit on resubmission based on HRQoL and safety data from an RCT	V: no therapeutic improvement IV: minor therapeutic improvement on resubmission based on data from an RCT and demonstrated PFS benefit
Durvalumab	II: considerable added benefit	III: moderate therapeutic improvement
Acalabrutinib	III: minor added benefit	V: no therapeutic improvement
Trastuzumab deruxtecan	II: considerable added benefit	V: no therapeutic improvement

Note: Roman numerals reflect HTA rating scales: I = major benefit to V = no benefit. HRQoL = health-related quality of life; HTA = health technology assessment; PFS = progression-free survival; RCT = randomized controlled trial.

**Table 5:** Overview of HTA recommendations from the first submission.

Medicine	Canada	England	Germany	France	Italy	Spain
Olaparib	**Negative**	**Restrictions**	**Restrictions**	**Positive**	**Restrictions**	**Positive**
Osimertinib	**Positive**	**Restrictions**	**Negative**	**Positive**	**Positive**	**Positive**
Durvalumab	**Positive**	**Restrictions**	**Positive**	**Positive**	**Positive**	**Restrictions**
Acalabrutinib	**Restrictions**	**Restrictions**	**Restrictions**	**Restrictions**	**Positive**	**Positive**
Trastuzumab deruxtecan	**Not submitted**	**Restrictions**	**Positive**	**Positive**	**Not submitted**	**Negative**

HTAs for olaparib in Canada and osimertinib in Germany were subsequently accepted with restrictions when confirmatory Phase III data (SOLO-2 and AURA3, respectively) were available.

**Table 6:** Lost clinical survival benefit.

Medicine	Canada	England	Germany	France	Italy	Spain
Number of patients affected						
**Trastuzumab deruxtecan**	3548	227	4233	5966	13,986	7869
**Acalabrutinib**	869	127	155	4641	1432	899
**Durvalumab**	2655	252	488	8311	3492	5577
**Osimertinib**	740	894	3342	4838	2174	2369
**Olaparib**	789	861	464	2144	1247	504
Overall mean *	2836					
**OS-derived life years lost (years)**						
**Trastuzumab deruxtecan**	3755	240	4480	6314	14,802	8328
**Acalabrutinib**	N/A	N/A	N/A	N/A	N/A	N/A
**Durvalumab**	4071	387	748	12,744	5354	8551
**Osimertinib**	265	320	1198	1734	779	849
**Olaparib**	848	926	499	2305	1341	542
Overall mean *	3391					
**PFS-derived life years lost (years)**						
**Trastuzumab deruxtecan**	3223	206	3845	5419	12,704	7147
**Acalabrutinib**	N/A	N/A	N/A	N/A	N/A	N/A
**Durvalumab**	2500	238	459	7826	3288	5252
**Osimertinib**	351	424	1588	2298	1033	1125
**Olaparib**	894	976	526	2430	1414	572
Overall mean *	2739					

* The overall average represents the mean of all numeric entries in the table across medicines and countries. DCO = data cut-off; N/A = not applicable; OS = overall survival; PFS = progression-free survival. Median OS and median PFS were not reached at the time of regulatory filing or latest DCO for acalabrutinib; therefore, acalabrutinib was excluded from the analysis.

**Table 7:** Scenario analysis assuming access within 6 months of regulatory approval *.

Metric (Across Cohorts)	Base Case	Scenario Analysis	Change from Base Case	Reduction in Loss (%)
**Market access approval to product listing, months**	17.7	4.8	12.9	72.9
**Patients without access, n**	2836	765	2071	73.0
**OS-derived life-years lost, n**	3391	934	2457	72.5
**PFS-derived life-years lost, n**	2739	722	2017	73.6

* Where subtracting 6 months would yield an implausible interval, a minimum lag of 1 month was applied for calculation purposes. This scenario reflects access within 6 months (not immediate access as in Germany), so losses are reduced but not eliminated. OS = overall survival; PFS = progression-free survival.

**Table 8:** Perspectives in decision-making.

Perspective	Impact of Market Access Process on the Stakeholder	Potential Biases That May Influence Decision-Making
Patients	Patients are unable to benefit from medicines ∘ A mean of 3391 OS-derived life-years were lost per medicine per country Access times may feel prolonged because patients may expect access when clinical trial results are published Prolonged regulatory and market access processes, with resulting revenue impacts, may discourage small health technology developers from launching medicines in some countries * (e.g., pharmaceutical companies)	N/A
Health technology companies	Small developers may struggle to survive prolonged times to revenue generation Large developers may delay submissions until more mature data are available, reducing uncertainty and strengthening price negotiations Small companies may be unable to do so, weakening their negotiation power Multiple market access processes may require developers to: ∘ Bring in external expertise, increasing costs ∘ Sell their health technology to a larger company ∘ Avoid launching their health technology in markets with different processes	Sunk cost bias: seeking return on significant investment over long-term R&D Urgency and loss-aversion bias: ∘ Irrecoverable revenue loss ∘ Need to deliver return on investment during patent life ∘ Risk of delay may affect launch decisions Overconfidence bias for medicines with substantial investment but immature data
Payers/HTA agencies	Healthcare systems must allocate limited budgets to new health technologies that provide the best value for money despite uncertainty	Status quo bias: payers may perpetuate the current state when managing finite budgets Loss-aversion bias: opportunity cost of investing in medicines with uncertain or modest benefit to society Information bias driven by the requirement for certainty before decision-making
Regulators	N/A	Urgency bias: delayed access perpetuates unmet clinical need for certain populations and diseases
Clinicians	Inability to prescribe new medicines, limiting treatment options, affecting patient outcomes, and creating uncertainty in clinical decision-making	N/A

HTA = health technology assessment; N/A = not applicable; OS = overall survival; R&D = research and development. * The term “health technology developer” (e.g., pharmaceutical companies) is used as defined in the European Union HTA Regulation, referring to innovative developers of new medicines rather than vendors or service providers.

## Data Availability

Data sources included published articles for clinical efficacy data (references are included in the manuscript) and published European Public Assessment Reports (EMA) as well as Health Canada’s Summary Basis of Decision for regulatory assessments of clinical benefit–risk balance. Epidemiology data were obtained from DR/Decision Resources, LLC (© 2025, all rights reserved; reprinted with permission). Payer assessments of clinical benefit–risk balance and regulatory approval dates were sourced from the IQVIA HTA Accelerator disease database (www.iqvia.com/landing/hta-accelerator, 13 October 2025). Reimbursement listing dates were extracted from the NAVLIN database (https://data.navlin.com, 13 October 2025). Additional information on data sources and epidemiology data is available in the Supplementary Materials of this manuscript. The Microsoft Excel file generated for this analysis is not available online. However, all data sources used in this work are publicly accessible as detailed above, and the calculations used to generate the data presented in the manuscript are described in Section 2 (Methods). This work was based on data retrieved via paid access from the IQVIA and NAVLIN databases. Regulatory approval dates can be obtained from national regulatory authority websites. Product listing dates can also be requested directly from national reimbursement agencies.

## Supplementary Materials

The following supporting information can be downloaded at: https://www.mdpi.com/article/10.3390/jmahp14020025/s1. Table S1: Overview of the medicines evaluated in the study: indications at first major and supporting trials [6,7,8,9,10,11,12,13,15,16,17,18,19,20,21,22,23,25,38,39,40,41,42,43,44,45,46,47,48]. Table S2: Market access dates and sources. Table S3: NAVLIN data sources for reimbursement listing dates. Table S4: Time to patient access for the five medicines in the six countries. Table S5: Country-specific annual incidence of eligible patients for the medicines included in the analysis.

## Abbreviations

The following abbreviations are used in this manuscript:

EMA	European Medicines Agency
HRQoL	Health-Related Quality of Life
HTA	Health Technology Assessment
JCA	Joint Clinical Assessment
MAIC	Matching Adjusted Indirect Comparison
mOS	Median Overall Survival
mPFS	Median Progression-Free Survival
OS	Overall Survival
PFS	Progression-Free Survival
SAT	Single Arm Trial
