# Supplementary material for: Impact of Market Access Delays on Time to Patient Access: Multi-Country Comparative Analysis Assessing the First Commercial Launch Indications for Five Oncology Medicines Across Europe and Canada
Source: J Mark Access Health Policy. 2026 Apr 28;14(2):25. doi: 10.3390/jmahp14020025 (PMC13214782; doi:10.3390/jmahp14020025)
Supplement: Supplementary file 1 [file jmahp-14-00025-s001.zip › jmahp-4116898-supplementary.pdf]

Impact of Market Access Delays on Time to Patient Access: Multi-Country Comparative Analysis Assessing the First Commercial Launch Indications for Five Oncology Medicines Across Europe and Canada

Authors

Barry Crean[1], David Parry[2], Alison Horsfield[2], James Ryan[2] and Nektarios Oraiopoulos[3]

Affiliations

[1] Pharmaceutical Sciences, R&D, AstraZeneca, Cambridge, CB2 0AA, UK

[2] Oncology Market Access and Pricing, Oncology Business Unit, AstraZeneca, Cambridge, CB2 1RY, UK

[3] Cambridge Judge Business School, University of Cambridge, CB2 1AG, UK

Corresponding Author

Barry Crean, Barry.Crean@astrazeneca.com

Supplementary Materials

Table S 1. Overview of the medicines evaluated in the study: indications at first major and supporting trials.

Medicine	First Major Launch Indication	Trial Name, NCT Number, Primary Completion Date, Source	Trial Population	Study Design and Phase	Number of Patients	Control Arm	mOS and mPFS,* Months, Versus Comparator (When Applicable)
Olaparib	EMA: maintenance treatment of adult patients with platinum-sensitive relapsed high-grade epithelial ovarian, fallopian tube, or primary peritoneal cancer who are in response (complete or	Study 19 [6-8,39] (first launch) NCT00753545, 30 Jun 2010, https://clinicaltrials.gov/study/NCT00753545	Adult patients with histologically diagnosed relapsed high-grade serous or high-grade endometrioid ovarian, primary peritoneal, and/or fallopian tube cancer	Phase II randomized	265	Placebo	ITT population: mOS = 29.8 vs 27.8 mPFS = 8.4 vs 4.8<br><br>BRCA mutation subgroup: mOS = 34.9 vs 31.9 mPFS = 11.2 vs 4.3

Medicine	First Major Launch Indication	Trial Name, NCT Number, Primary Completion Date, Source	Trial Population	Study Design and Phase	Number of Patients	Control Arm	mOS and mPFS,* Months, Versus Comparator (When Applicable)
	partial) to platinum-based chemotherapy [38]<br><br>Canada: maintenance treatment of adult patients with platinum-sensitive relapsed *BRCA*-mutated high-grade epithelial ovarian, fallopian tube, or primary peritoneal cancer who are in response (complete or partial) to platinum-based chemotherapy [12]	SOLO-2<br>5-year follow-up [9,10]<br>NCT01874353, 19 Sep 2016, https://clinicaltrials.gov/study/NCT01874353	Adult patients with *BRCA*-mutated, relapsed, advanced, high-grade serous or endometrioid ovarian cancer with a complete or partial clinical response after platinum-based chemotherapy	Phase III randomized	295	Placebo	mOS = 51.7 vs 38.8<br>mPFS = 19.1 vs 5.5
Osimertinib	Europe: treatment of patients with T790M mutation-positive NSCLC, irrespective of	AURAex [11,16]<br>(first launch)<br>NCT01802632, 1 May 2015, https://clinicaltrials.gov/study/NCT01802632	Adult patients with advanced NSCLC who have already received at least	Phase I/II single-arm	201 T790M	None	mOS = NR<br>mPFS = 11

Medicine	First Major Launch Indication	Trial Name, NCT Number, Primary Completion Date, Source	Trial Population	Study Design and Phase	Number of Patients	Control Arm	mOS and mPFS,* Months, Versus Comparator (When Applicable)
	previous treatment with an EGFR-TKI [40]<br><br>Canada: treatment of patients with locally advanced or metastatic EGFR T790M mutation-positive NSCLC who have progressed on or after EGFR-TKI therapy [41]		one course of specific anticancer treatment				
		AURA2 [12]†<br>NCT02094261, 15 Apr 2016,<br>https://clinicaltrials.gov/study/NCT02094261	Adult patients with a confirmed diagnosis of EGFR-positive and T790M-positive NSCLC, who have progressed following prior therapy with an approved EGFR-TKI	Phase II single-arm	210 T790M	None	mOS = NR<br>mPFS = 9.9
		AURA3 [13,15]†<br>4-year follow-up<br>NCT02151981, 15 Apr 2016,<br>https://clinicaltrials.gov/study/NCT02151981		Phase III randomized	419 T790M	SoC	mOS = 26.8 vs 22.5<br>mPFS = 10.1 vs 4.4<br>mPFS in IMPRESS [49] (CT, synthetic control) = 5.4
Durvalumab	Europe and Canada: monotherapy for the treatment of locally advanced, unresectable NSCLC in adults whose tumors express PD-L1 on	PACIFIC [17-19]<br>first launch and follow-up [19]<br>NCT02125461, 13 Feb 2017,<br>https://clinicaltrials.gov/study/NCT02125461	Adult patients with locally advanced, unresectable NSCLC (stage III) who have not progressed following definitive,	Phase III randomized	713	Placebo	Primary:<br>mOS = 17.2 vs 5.6<br>mPFS = 16.8 vs 5.6<br><br>5-year follow-up:<br>mOS = 47.5 vs 29.1<br>mPFS = 16.9 vs 5.6

Medicine	First Major Launch Indication	Trial Name, NCT Number, Primary Completion Date, Source	Trial Population	Study Design and Phase	Number of Patients	Control Arm	mOS and mPFS,* Months, Versus Comparator (When Applicable)
	≥ 1% of tumor cells and whose disease has not progressed following platinum-based chemoradiation therapy [42,43]		platinum-based, concurrent chemoradiation				
Acalabrutinib	Europe and Canada: treatment of adult patients with CLL, as monotherapy or in combination with obinutuzumab in the first-line setting and as monotherapy for relapsed/refractory disease [44,45]	ELEVATE-TN [20-22,46] first launch and follow-up NCT02475681, 8 Feb 2019, https://clinicaltrials.gov/study/NCT02475681	Treatment of adult patients with previously untreated CLL, as monotherapy or in combination with obinutuzumab	Phase III randomized	535	SoC	mOS = NE<br>mPFS = NE
Trastuzumab deruxtecan	Europe and Canada: monotherapy for the treatment of adult patients with unresectable or metastatic HER2-	DESTINY-Breast01 [25] (first launch) NCT03248492, 21 Mar 2019, https://clinicaltrials.gov/study/NCT03248492	Adult patients with HER2-positive, unresectable, and/or metastatic breast cancer	Phase II single-arm	184	None	mOS = 24.6 vs 12.6<br>mPFS = 16.4 vs 3.7
		DESTINY-Breast02 [23] (first launch)		Phase III randomized	599	SoC	mOS = 39.2 vs 26.5<br>mPFS = 17.8 vs 6.9

Medicine	First Major Launch Indication	Trial Name, NCT Number, Primary Completion Date, Source	Trial Population	Study Design and Phase	Number of Patients	Control Arm	mOS and mPFS,* Months, Versus Comparator (When Applicable)
	positive breast cancer who have received one or more prior anti-HER2-based regimens [47,48]	NCT03523585, 30 Jun 2022, https://www.clinicaltrials.gov/study/NCT03523585	previously treated with trastuzumab emtansine				

BRCA = breast cancer gene; CLL = chronic lymphocytic leukemia; CT, chemotherapy; EGFR = epidermal growth factor receptor; EMA = European Medicines Agency; HER2 = human epidermal growth factor receptor 2; ITT = intention-to-treat; MAIC = matching-adjusted indirect comparison; mOS = median overall survival; mPFS = median progression-free survival; NCT = National Clinical Trial; NE = not evaluable; NR = not reached; NSCLC = non-small cell lung cancer; ORR = overall response rate; PD-L1 = programmed death ligand-1; SoC = standard of care; TKI = tyrosine kinase inhibitor. *Primary endpoints were met in all trials. More details on how all efficacy endpoints were assessed in each trial can be found in the source studies referenced in this table and on the https://www.clinicaltrials.gov/ webpages of each trial linked in this table. †The data used in this study to calculate OS and PFS benefit is from a pooled analysis of AURA2 and AURAex (mOS = NR and mPFS = 4.5; see **Error! Reference source not found.**).

Table S 2. Market access dates and sources.

Date Source		IQVIA HTA Accelerator Database	IQVIA HTA Accelerator Database	IQVIA HTA Accelerator Database	IQVIA HTA Accelerator Database	NAVILN Database	NA	NA	NA	Astra Zeneca
**Drug name**	**Country**	**Regulatory approval date**	**HTA process start date**	**HTA recommendation date**	**HTA recommendation**	**Product listing date**	**Regulatory approval to HTA recommendation time (months)**	**HTA start to recommendation duration (months)**	**Regulatory approval to product listing time (months)**	**Funded Early Access Program**
Acalabrutinib	England	05-Nov-20	06-Mar-20	21-Apr-21	Positive with restrictions	06-Dec-20	6	14	1	No
Acalabrutinib	Germany	05-Nov-20	01-Dec-20	05-Aug-21	Positive with restrictions	*01-Dec-20*	9	8	1	No
Acalabrutinib	France	05-Nov-20	24-Mar-21	05-May-21	Positive with restrictions	02-Apr-23	6	1	29	Yes
Acalabrutinib	Canada	08-Jan-20	07-Apr-20	17-Nov-20	Positive with restrictions	17-Nov-20	10	7	10	No
Acalabrutinib	Italy	05-Nov-20	20-Nov-20	03-Dec-21	Positive	14-Dec-21	13	13	13	No
Acalabrutinib	Spain	05-Nov-20	03-Dec-20	26-Jan-22	Positive	26-Jan-22	15	14	15	No
Trastuzumab deruxtecan	England	16-Feb-21	14-Aug-20	26-May-21	Positive with restrictions	23-Mar-21	3	10	1	Yes
Trastuzumab deruxtecan	Canada	15-Jun-22	N/A	28-Sep-22	N/A	*31-Aug-24*	N/A	N/A	27	No
Trastuzumab deruxtecan	France	08-Feb-21	21-Apr-21	16-Jun-21	Positive	23-Feb-24	4	2	37	Yes
Trastuzumab deruxtecan	Italy	08-Feb-21	N/A	N/A	N/A	*31-Aug-24*	N/A	N/A	43	No
Trastuzumab deruxtecan	Germany	08-Feb-21	01-Aug-22	02-Feb-23	Positive	01-Feb-22	24	6	12	No
Trastuzumab deruxtecan	Spain	08-Feb-21	14-Jun-21	07-Jul-22	Negative	*31-Aug-24*	17	13	43	No
Durvalumab	Germany	21-Sep-18	15-Oct-18	04-Apr-19	Positive	15-Oct-18	7	6	1	No
Durvalumab	Italy	21-Sep-18	01-Mar-19	06-Sep-19	Positive	01-May-19	12	6	7	No
Durvalumab	England	21-Sep-18	01-Mar-18	01-May-19	Positive with restrictions	12-Oct-18	7	14	1	Yes
Durvalumab	France	21-Sep-18	23-Jan-19	06-Feb-19	Positive	24-Apr-20	5	0	19	Yes
Durvalumab	Canada	04-May-18	21-Sep-18	03-May-19	Positive	26-Mar-19	12	7	11	No
Durvalumab	Spain	21-Sep-18	14-Dec-18	31-Jan-20	Positive with restrictions	31-Jan-20	17	14	17	No

Olaparib	Canada	29-Apr-16	01-Apr-16	28-Sep-17	Negative	31-Oct-17	17	18	18	No
Olaparib	France	16-Dec-14	15-Apr-15	03-Jun-15	Positive recommendation	24-Jan-18	6	2	38	Yes
Olaparib	Germany	16-Dec-14	01-Jun-15	27-Nov-15	Positive with restrictions	01-Jun-15	12	6	6	No
Olaparib	Spain	16-Dec-14	24-Mar-15	21-Jan-16	Positive	21-Jan-16	13	10	13	No
Olaparib	England	16-Dec-14	18-Nov-14	27-Jan-16	Positive with restrictions	27-Jan-16	14	15	14	Yes
Olaparib	Italy	16-Dec-14	17-Dec-14	31-Aug-16	Positive with restrictions	27-Aug-16	21	21	21	No
Olaparib	Canada	29-Apr-16	01-Apr-16	17-Aug-17	Positive with restrictions	17-Aug-17	16	17	16	No
Osimertinib	Germany	02-Feb-16	15-Mar-16	15-Sep-16	Negative	15-Mar-16	8	6	1	No
Osimertinib	England	02-Feb-16	27-Jan-16	26-Oct-16	Positive with restrictions	30-Oct-16	9	9	9	Yes
Osimertinib	France	02-Feb-16	08-Jun-16	21-Sep-16	Positive	26-Jul-19	8	4	42	Yes
Osimertinib	Italy	02-Feb-16	01-Apr-16	08-Aug-17	Positive	14-Aug-17	18	16	19	No
Osimertinib	Canada	05-Jul-16	01-Apr-16	04-May-17	Positive	01-Jun-17	10	13	11	No
Osimertinib	Germany	02-Feb-16	01-May-17	19-Oct-17	Positive	14-Nov-17	21	6	22	No
Osimertinib	Spain	02-Feb-16	04-Mar-16	03-Aug-18	Positive	03-Aug-18	30	29	30	No

Note 1 month assumed to be 30 days. 31 August 2024 was the latest product listing date in cohort (France) and taken as a proxy product listing date for Tratuzumab deruxtecan in Canada, Italy and Spain to enable calculations. Two rows shown for olaparib in Canada and osimertinib in Germany related to initial single-arm trial HTA submission followed by a second submission with RCT data. Access related calculations were derived using the second HTA recommendation date.

HTA = health technology assessment.

Table S 3. NAVLIN data sources for reimbursement listing dates.

Country	Source
France	Health Insurance Fund
Germany	Lauer-taxe
Italy	Codifa
Spain	Botplus
United Kingdom	National Health Service Business Services Authority
Canada – Newfoundland & Labrador	Newfoundland & Labrador Health and Community Services
Canada – Ontario	Exceptional Access Program, Ministry of Health and Long-Term Care
Canada – Quebec	Board of Health Insurance Quebec (Régie de l’assurance maladie du Québec)
Canada – Yukon	Yukon Drug Formulary
Canada – Alberta	Interactive Drug Benefit List, Alberta
Canada – Manitoba	Manitoba Drug Formulary
Canada – Saskatchewan	The Saskatchewan Drug Formulary

Table S 4. Time to patient access for the five medicines in the six countries.

Medicine	Canada	England	Germany	France	Italy	Spain	Mean
**Time between regulatory approval and reimbursement listing date (months)**							
Trastuzumab deruxtecan*	27	1	12	37	43	43	17.73
Acalabrutinib	10	1	1	29	13	15	
Durvalumab	11	1	1	19	7	17	
Osimertinib	11	9	22	42	19	30	
Olaparib	18	14	6	38	21	13	
**Time between regulatory approval and HTA benefit assessment outcome (months)**							
Trastuzumab deruxtecan*	N/A	3	24	4	N/A	17	12.34
Acalabrutinib	10	6	9	6	13	15	
Durvalumab	12	7	7	5	12	17	
Osimertinib	10	9	21	8	18	30	
Olaparib	17	14	12	6	21	13	
**Time between HTA submission to HTA benefit assessment outcome (months)**							
Trastuzumab deruxtecan*	N/A	10	6	2	N/A	13	10.15
Acalabrutinib	7	14	8	1	13	14	
Durvalumab	7	14	6	0	6	14	
Osimertinib	13	9	6	4	16	29	
Olaparib	18	15	6	2	21	10	

*Trastuzumab deruxtecan was not launched in Canada, Italy and Spain.

HTA = health technology assessment; N/A = not applicable.

Table S 5. Country-specific annual incidence of eligible patients for the medicines included in the analysis.

Drug name	Country incidence (eligible patients/ year)					
	Canada	England	Germany	France	Italy	Spain
Trastuzumab deruxtecan	1581	2332	4257	1935	3873	2179
Acalabrutinib	997	1470	2148	1903	1276	724
Durvalumab	2932	4325	7314	5150	5663	4040
Osimertinib	805	1187	1848	1371	1400	934
Olaparib	516	762	1000	680	724	453
